# Is Long Term Creatine and Glutamine Supplementation Effective in Enhancing Physical Performance of Military Police Officers?

**DOI:** 10.2478/hukin-2014-0098

**Published:** 2014-11-12

**Authors:** Celismar Lázaro da Silveira, Thiago Siqueira Paiva de Souza, Gilmário Ricarte Batista, Adenilson Targino de Araújo, Júlio César Gomes da Silva, Maria do Socorro Cirilo de Sousa, Carlos Marta, Nuno Domingo Garrido

**Affiliations:** 1 Department of Sport Sciences, University of Trás-os-Montes and Alto Douro, Vila Real, Portugal.; 2 Research Centre for Sport, Health and Human Development, Vila Real, Portugal.; 3 Kinanthropometry and Human Development Laboratory - LABOCINE -UFPB, João Pessoa / PB, Brazil.; 4 Associate Programme on Graduate Program in Physical Education UPE / UFPB, João Pessoa, Paraíba, Brazil.; 5 Department of Sport Sciences, Polytechnic Institute of Guarda (IPG, Guarda, Portugal).; 6 Research Unit for Inland Development (UDI, Portugal).

**Keywords:** supplementation, exercise, creatine, glutamine, RCT, military police officers

## Abstract

The objective of this study was to analyze the effect of supplementation with creatine and glutamine on physical fitness of military police officers. Therefore, an experimental double blind study was developed, with the final sample composed by 32 men randomly distributed into three groups: a group supplemented with creatine (n=10), glutamine (n=10) and a placebo group (n=12) and evaluated in three distinct moments, in an interval of three months (T1, T2 and T3). The physical training had a weekly frequency of 5 sessions × 90 min, including strength exercises, local muscular resistance, flexibility and both aerobic and anaerobic capacity. After analyzing the effect of time, group and interaction (group × time) for measures that indicated the physical capabilities of the subjects, a significant effect of time for the entire variable was identified (p<0,05). However, these differences were not observed when the univaried intragroups and intergroups analysis was performed (p>0,05). In face of the results it was concluded that supplementation with creatine and glutamine showed no ergogenic effect on physical performance in military police officers.

## Introduction

Using nutritional supplements to increase physical performance is, nowadays, a much highlighted strategy in physically active individuals (Maughan and Burke, 2004). Among the numerous supplements commercialized in the market today, it is possible to find creatine (Cr) and glutamine (Gl), which had their ergogenic action tested and were proved to increase levels of muscular strength and power along with resistance training (Wright et al., 2007; Terjung et al., 2000; Bemben and Lamont, 2005; Gualano et al., 2008).

Although data about long term supplementation is scarce (particularly in athletes), the literature refers to several well controlled research projects, indicating that supplementation with creatine (ACSM, 2000; Kreider, 1997; Volek et al., 1999) and glutamine (Antonio et al., 1999; Ziegler et al., 1990) is safe, as long as done carefully and by professionals.

Although many studies were performed with these supplements, it is still not clear if the long term use of glutamine or creatine affects positively protein synthesis, body composition, anaerobic power and other physiological variables (Fontana, 2006).

The monohydrated creatine (Crm) is widely used, above all, by athletes and physically active individuals, due to its possible ergogenic effects on muscular mass and anaerobic physical performance (Mendes et al., 1999; Mihic et al., 2000). Therefore, it is suggested that an increase in muscular concentration of total creatine (CrT) and phosphocreatine (CP), induced by supplementation with monohydrate creatine (Crm), can increase the availability of CP and, consequently, accelerate the rate of re-synthesis of ATP during intermittent anaerobic exercise, enhancing physical performance in this type of exercise. (Balsom et al., 1995; Febbraio et al., 1995; Maganaris and Maughan, 1998).

Glutamine is a crucial amino acid for the human body, with functions that include its use as fuel for the cells of the immune system, together with isoleucine, valine and leucine being more abundant in muscle tissue, having bigger energetic and metabolic importance (Ceddia et al., 2000; Newsholme et al., 2003).

The literature indicates that glutamine is efficient in increasing the absorption of electrolytes and water in animals (Nath et al., 1992; Van Loon et al., 1996). Both glutamine and alanine, important amino acids for the human body, have been proved efficient for antioxidant defense during serious diseases (Abilés et al., 2008; Kumar and Anandan, 2007). Glutamine has been reclassified from dispensable amino acids to a conditionally indispensable amino acid for the homeostatic functions of the organism (Rogero and Tirapegui, 2003).

Based on the aforementioned studies, according to Military Physical Training (MPT) which is an exercise program that has a principle of specificity and that seeks to improve the physical qualities that are most sought in the labor activities of this profession, in which muscular strength and endurance of the individual are prerequisites for efficient performance. Currently, ergogenic means can be used with police students to minimize fatigue caused by high volume of training and improve their physical fitness. This study examined the effect of supplementation with creatine and glutamine on physical performance of military police officers.

## Material and Methods

### Participants

The research was an experimental randomized control trial, with a non probabilistic sample process and longitudinal approach. Military police officers from the Estate of Tocantins-Brazil, physically active and apparently healthy, participated in the study. The research applied a double blind model with random distribution of subjects to the creatine, glutamine and placebo groups.

In order to select the sample we used a calculation based on the mistake of estimation α by 5%, estimative of the size of the effect of 0,5, the power β of 86% and level of reliability by 95% through the statistic software Gpower, performing a number of 36 subjects, who were randomly divided into three groups: one that received supplementation with creatine (GCr, n=12), other with glutamine (GGL, n=12) and a placebo group (GPL, n=12).

All the participants signed the Term of Free and Clarified Consent, after being informed about the procedures of the research. The study was approved by the Committee of Ethics in Research, registered under the number 0312/2011, in accordance with the ethical principles contained in the Declaration of Helsinki

To be included in the study the following criteria were established: age between 18 and 30; non smoking; no alcohol drinking; no anabolic steroids use; no nutritional supplements of any kind; moreover, the participants had to answer negatively to all the items of the *Physical Activity Readiness Questionnaire* (PAR-Q).

The final sample was composed by 32 subjects (GCr= 10; GGL= 10; GPL=12), the others were excluded for not being able to perform the physical tests (n=2), and for being absent in the days of the evaluations due to health problems (n=2).

### Procedures

The evaluations were performed on premises of the Military Police Academy. The groups were evaluated in three distinct moments, within a three months period: at the beginning of the activities (T1); after six weeks of activities (T2); and after twelve weeks of activities (T3).

MPT was performed five times per week with duration of 90 min. Each training session included three parts: a warm up and stretching; physical exercises; and a cool down. The exercises were a mix of neuromuscular and cardiopulmonary training. The activities were supervised by specialists and totally fulfilled under the same military context. Supplementation was given under the supervision of a nutritionist. The creatine and glutamine supplementation protocols were based on previously confirmed scientific data (Cox et al., 2002; Deutekom et al., 2000; Edwards et al., 2000; Okudan and Gokbel, 2005).

The supplements of glutamine, creatine and placebo (cornflour) were given orally, dissolved in a medium sweet liquid (150 to 300 ml). The adaptation dose (first week) from 0,3 g·kg^−1^, was divided into three equal daily doses distributed along the day, and the maintenance dose (about 12 weeks) by 0,03 g·kg^−1^ in a unique dose, 30 min after MPT. All the supplements were given in sachets. The ingestion of the supplements took place in the morning at 6:30 am, before the beginning of the MPT activities, for 93 days without interruption.

### Measures

To verify the effect of supplementation on physical performance of military policemen, as well as aerobic and anaerobic capacity, muscular strength of upper and lower limbs, flexibility and local muscle endurance tests were used.

The aerobic capacity was evaluated by the 12 minute Cooper test (1968); the anaerobic capacity was evaluated by the shuttle run test; muscle strength of the upper limbs was evaluated by pushups (Mayhew, 1991); and muscle strength of the lower limbs was evaluated by the horizontal jump (Scott and Docherty, 2004); flexibility was determined by the sit and reach test (ACSM, 2010); and abdominal muscle endurance was evaluated by the sit-up test (Sarti et al., 1996). In order not to compromise the study reliability, physical tests were performed by only one researcher.

### Statistical Analysis

Data were analysed by the SPSS for Windows version 20.0. The values were expressed as means and standard deviation (SD). Before performing the inferential analysis, for numeric variables, the assumption of homogeneity was tested by the Levene’s test and the sphericity was tested by the Mauchly’s test whereas for the absence of the sphericity the correction of Greenhouse-Geiser was adopted. An Anova one way, with the Tuckey HSD post hoc test, was used to check possible differences among the groups, for each phase of the data collection, as well as the possible alterations between the measures. A two way Anova was performed to check the time effect (measures: T1 × T2 × T3), the group effect (placebo × creatine × glutamine) and the time × group interaction, for the variables of physical performance. Additionally the respective delta of variation (% Δ) was presented between the first and the second (% Δ1), and between the second and the third (% Δ2) measurements. The effect size was calculated based on Cohen’s (1988) criteria. It was considered small if 0≤ldl≤0.2, medium if 0.2≤ldl≤0.5, and large if ldl>0.5. The level of significance was set at α=0.05.

## Results

Table 1 presents the multivariated analysis of the variable of physical performance. A significant effect was identified in time for the entire variable analysed. Table 2 presents the descriptive and univariate analysis of the variables of physical performance in the groups GCr, GGL and GPL. The aerobic capacity (VO2) presented differences between T1 vs T2 and T1 vs T3, mainly from the T1 for the T2, for the GPL (% Δ1 = 13) and GGL (% Δ1 = 12), given the measures were different for the GPL (F = 8,657, p = 0,001), with alterations between T1 vs T2 (p = 0,002) and T1 vs T3 (0,004), and for the GGL (F = 5,623, p = 0,009), with significant post hoc for T1 vs T2 (p = 0,018) and T1 vs T3 (p= 0,021).

The anaerobic capacity (Shuttle-Run), presented differences in the following times: GPL (F = 8,109, p = 0,001, post hoc T1 vs T2 p = 0,018 and T1 vs T3 p = 0,001); for the GCr (F = 19,791, p = 0,001, post hoc T1 vs T2 p = 0,001 and T1 vs T3 p = 0,001), and for the GGL (F = 27,088, p = 0,001, post hoc T1 vs T2 p = 0,001 and T1 vs T3 p = 0,001).

Muscle endurance of the abdomen (RML_ABD) presented differences in the following measurements points: GPL (F = 47,185, p = 0,001; post hoc T1 vs T2, p = 0,001; T1 vs T3, p= 0,001), GCr (F = 44,738, p = 0,001; post hoc T1 vs T2, p = 0,001; T1 vs T3, 0,001) GGL (F = 29,533, p = 0,001; post hoc T1 vs T2, p = 0,001; T1 vs T3, p= 0,001).

Muscle strength (MS) presented differences in the following measurements points: GPL (F = 30,984, p = 0,001; post hoc T1 vs T2, p = 0,015; T1 vs T3, p = 0,001; T2 vs T3, p = 0,001). GCr (F = 58,391, p = 0,001; post hoc T1 vs T2, p = 0,001; T1 vs T3, p = 0,001; T2 vs T3, p = 0,001); GGL (F = 9,664, p = 0,001; post hoc T1 vs T2, p= 0,001).

Muscle strength (MS) and flexibility did not show differences between times. For the between group analysis there were no differences for the measures of T1, T2 and T3, which did not indicate effect of the group, as well as the interaction among groups and time.

## Discussion

The main objective of this study was to evaluate the ergogenic effects of creatine and glutamine supplementation on physical performance of military police officers. All tests and measurements, sample implementation and internal consistency were high, and performed according to protocols validated by the literature. The follow-up period (three months), may not have caused the expected effects of the supplementary intervention on the dependent variables. The one which presented a high size effect (η^2^>0.80), was aerobic capacity (VO_2_).

It was proposed as the hypothesis of this study that supplementation with creatine and glutamine would improve physical performance of military officers after three months of physical training, as muscle strength and power benefit with the consumption of these supplements (Wright et al., 2007; Terjung et al., 2000; Bemben and Lamont, 2005; Gualano et al., 2008). However, in this study, supplementation with creatine and glutamine did not confirm an ergogenic effect, as there were no significant differences observed in the two groups taking supplements.

These results corroborate with the findings of Bemben and Lamont (2005) which examined American Football athletes during 9 weeks of training using fartlek, Interval training and plyometrics, and no significant changes between the experimental group (using creatine) and the placebo group were observed. Furthermore, Fontana (2006), in a study with 32 male and healthy subjects, aged 21,7 ± 2,9 years, volunteers, divided into three groups (placebo, supplemented with glutamine and supplemented with creatine), after 8 weeks of training that consisted of 4 sessions per week lasting 1,5 h each, did not observe any differences among the groups in the following variables: maximum power, medium power and fatigue.

According to the study of Buck et al. (2003), with 32 military male subjects aged 19 to 26, divided into three groups (control, supplemented with creatine and supplemented with creatine and maltodextrin), who performed a series of physical activity drills, and it was observed that the dose of monohydrate creatine did not cause significant ergogenic effects on physical performance, represented by the following variables: explosive strength, local muscle strength and anaerobic power.

This suggests that military physical training (MPT) conducted in this study, was dependent on the anaerobic or aerobic power, especially taking into consideration that, according to Williams et al. (2000), there is no association between the improvement in performance of these components and supplementation with creatine. Based on the results of this work, it was concluded that supplementation with glutamine cannot be associated with the improvements in performance in dependent tasks of anaerobic and aerobic power.

However, supplementation with creatine seems to be more effective in endurance exercise, characterized by a high aerobic component, intermittent stimulus of high intensity and short duration (ACSM, 2010). Moreover, there are anaerobic moments in which the speed and intensity of the activity are increased, as at the end of competitions of longer duration when the increased reserve of muscular phosphocreatine can provide a better performance for the athlete. Such associations are observed in military physical training. Glutamine has been frequently associated with improvements of the immune system, reducing the frequencies of occurrence of infections in the respiratory tract.

## Conclusion

The three months supplementation with creatine and glutamine in MPT did not show ergogenic effects on the following variables: aerobic capacity, anaerobic capacity, muscular strength in the upper and lower limbs, flexibility and local muscle endurance.

Further research focusing on MPT is required. Therefore, it is necessary to include a longer period of physical preparation, quantity of repeated measures and standardization of activities which allows a better control of the intensity. The inclusion of the subjective scale of effort perception is suggested, thus allowing the assessment and control of the training program.

## Figures and Tables

**Table 1:** Multivaried analysis of the variables of the physical performance (n = 32)

	**VO2 (ml/kg/min)^‡^**			**Shuttle Run (s)^‡^**		
Statistic Effect	F/p-value^ε^	η**^2^**	*post hoc*	F/p-value	η**^2^**	*post hoc*
**Time (T)**	117,608^*^	0.80	T1≠T2 T1≠T3	115,421*	0.80	T1≠T2 T1≠T3 T2≠T3
**Group (G)**	0,446	0.03	-	0,144	0.01	-
**Interaction T × G**	3,612	0.20	-	0,628	0.04	-

	**SH _(cm)_^‡^**			**FB (rep)^‡^**		

Statistic Effect	F/p-value	η**^2^**	*post hoc*	F/p-value	η**^2^**	*post hoc*
**Time (T)**	10,847*	0.30	T1≠T2 T1≠T3	153,910*	0.80	T1≠T2 T1≠T3 T2≠T3
**Group (G)**	0,448 (p> 0,05)	0.03	-	0,984	0.05	-
**Interaction T × G**	0,188 (p> 0,05)	0.01	-	2,495	0.10	-

	**RML_ABD(rep)^‡^**			**Flexibility (cm)^‡^**		

Statistic Effect	F/p-value	η**^2^**	*post hoc*	F/p-value^ε^	η**^2^**	*post hoc*
**Time (T)**	308,132*	0.90	T1≠T2 T1≠T3 T2≠T3	37,254*	0.60	T1≠T2 T1≠T3 T2≠T3
**Group (G)**	0,904	0.05	-	0,693	0.04	-
**Interaction T × G**	1,600	0.10	-	0,746	0.04	-

VO_2 _– aerobic capacity; Shuttle Run – anaerobic capacity; RML_ABD – Abdominal muscle endurance; SH – strength of the lower limbs; FB – strength of the upper limbs.

* Variable that presented effect in the time;

ε The correction of Greenhouse-Geisserthe was adopted; p<0,05.

**Table 2:** Descriptive and univariated analysis of the variables of physical performance in the groups supplemented with creatine (CR, n = 10), glutamine (GL, n = 10) and placebo (PL, n = 12) before, during and after the intervention

	Beginning (T1) M ± DP	6 weeks (T2) M ± DP	12 weeks (T3) M ± DP	% Δ1 (T2–T1)	% Δ2 (T3–T2)
**VO_2 (ml/kg/min)_**					
Placebo	46,94±4,55^a,b^	53,18±3,9	52,82±3,87	13,3	−0,7
Creatine	48,26±4,46	52,15±4,05	51,27±3,43	8,1	−1,7
Glutamine	48,36±5,12	54,15±4,47	54,02±3,46	12,0	−0,2
**Shuttle Run _(s)_**					
Placebo	9,87±0,48 ^a,b^	9,39±0,37	9,22±0,42	−4,9	−1,8
Creatine	9,89±0,25 ^a,b^	9,26±0,35	9,15±0,24	−6,4	−1,2
Glutamine	9,91±0,28 ^a,b^	9,28±0,29	9,11±0,17	−6,4	−1,8
**RML_ABD _(rep)_**					
Placebo	38,91±5,79^a,b^	58,75±5,15	62,91±4,75	51,0	7,1
Creatine	41,6±4,74 ^a,b^	60,6±4,81	62,8±4,73	45,7	3,6
Glutamine	40,8±7,13 ^a,b^	56,20±4,58	60,1±5,82	37,7	6,9
**SH _(cm)_**					
Placebo	227,41±22,1	235±23,76	235,33±20,59	3,3	0,1
Creatine	223,1±14,65	229,9±15,57	229,3±17,0	3,0	−0,3
Glutamine	218,3±18,28	228,6±24,76	228,3±21,46	4,7	−0,1
**FB _(rep)_**					
Placebo	38,41±7,19^a,b, c^	47,41±6,73	62,16±8,36	23,4	31,1
Creatine	36,6±5,44 ^a,b, c^	48,6±3,65	56,7±3,09	32,8	16,7
Glutamine	42,2±10,43 ^b^	51,60±7,74	59,4±7,8	22,3	15,1
**Flexibility**					
Placebo	31,5±6,41	32,91±5,35	34,9±5,59	4,5 6,1	
Creatine	34,4±6,71	36,2±6,3	37,9±7,12	5,2	4,7
Glutamine	31,5±9,72	31,8±9,54	34,9±8,76	1,0	9,7

Vo2, - aerobic capacity; Shuttle Run – anaerobic capacity; RML_ABD – Abdominal muscular endurance; SH – strength of the lower limbs; FB – strength of the upper limbs; intragroups significant between:

a T1 e T2;

b T1 e T3;

c T2 e T3.
